# Mitochondrial dynamics modulate the allergic inflammation in a murine model of allergic rhinitis

**DOI:** 10.1002/iid3.1002

**Published:** 2023-09-22

**Authors:** Xu‐qing Chen, Long‐yun Zhou, Hua‐an Ma, Ji‐yong Wu, Shu‐fen Liu, Yong‐jun Wu, Dao‐nan Yan

**Affiliations:** ^1^ Department of Otolaryngology, Jiangsu Province Hospital of Chinese Medicine Affiliated Hospital of Nanjing University of Chinese Medicine Nanjing China; ^2^ Department of Rehabilitation Medicine, Jiangsu Province Hospital The First Affiliated Hospital of Nanjing Medical University Nanjing China; ^3^ Spine Disease Institute, Longhua Hospital Shanghai University of Traditional Chinese Medicine Shanghai China; ^4^ The First Clinical Medical College Nanjing University of Chinese Medicine Nanjing China

**Keywords:** allergic inflammation, allergic rhinitis, Mdivi‐1, mitochondrial dynamics

## Abstract

**Objective:**

Allergic rhinitis (AR) is a common allergic disorder, afflicting thousands of human beings. Aberrant mitochondrial dynamics are important pathological elements for various immune cell dysfunctions and allergic diseases. However, the connection between mitochondrial dynamics and AR remains poorly understood. This study aimed to determine whether mitochondrial dynamics influence the inflammatory response in AR.

**Methods:**

In the present study, we established a murine model of AR by sensitization with ovalbumin (OVA). Then, we investigated the mitochondrial morphology in mice with AR by transmission electron microscopy and confocal fluorescence microscopy, and evaluated the role of Mdivi‐1 (an inhibitor of mitochondrial fission) on allergic symptoms, inflammatory responses, allergic‐related signals, and reactive oxygen species formation.

**Results:**

There was a notable enhancement in mitochondrial fragmentation in the nasal mucosa of mice following OVA stimulation, whereas Mdivi‐1 prevented aberrant mitochondrial morphology. Indeed, Mdivi‐1 alleviated the rubbing and sneezing responses in OVA‐sensitized mice. Compared with vehicle‐treated ones, mice treated with Mdivi‐1 exhibited a reduction in interleukin (IL)‐4, IL‐5, and specific IgE levels in both serum and nasal lavage fluid, and shown an amelioration in inflammatory response of nasal mucosa. Meanwhile, Mdivi‐1 treatment was associated with a suppression in JAK2 and STAT6 activation and reactive oxygen species generation, which act as important signaling for allergic response.

**Conclusion:**

Our findings reveal mitochondrial dynamics modulate the allergic responses in AR. Mitochondrial dynamics may represent a promising target for the treatment of AR.

## Graphical abstract

Excessive mitochondrial fission was induced by ovalbumin (OVA) stimulation in a mouse model of allergic rhinitis (AR), whereas Mdivi‐1 (an inhibitor of mitochondrial fission) prevented aberrant mitochondrial morphology. Moreover, Mdivi‐1 attenuated the allergic inflammatory responses, allergic‐related signal activation, and reactive oxygen species generation in OVA‐sensitized mice. Our findings reveal mitochondrial dynamics modulate the allergic responses in AR.

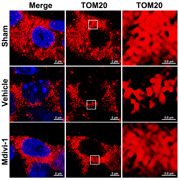

## 1 INTRODUCTION

Allergic rhinitis (AR) is a common allergic disorder, affecting individuals of all ethnic groups and all ages.^1^, ^2^ The prevalence of epidemiologic AR and pollen‐induced AR is 32.4% and 18.5%, respectively, in northern China.^3^ Worldwide, 1.14%–40.4% of individuals have clinical manifestations of AR.^4^, ^5^ AR affects social life and work, has high economic costs, and imposes heavy burdens on patients, their families, and society.^6^, ^7^ As such, AR represents a public health problem of significant concern.

AR primarily arises from exposure and sensitization to specific allergens. Initial sensitization by allergens can lead to T helper type 2 (Th2) cell differentiation, release of Th2 cytokines and overproduction of immunoglobulin E (IgE). Subsequently, immune cells are activated to release cytokines and chemical mediators following allergen re‐exposure, leading to hemangiectasis, mucosal edema, and allergic inflammation in the nose lining.^8^ Among the complicated pathological elements associated with AR, mitochondrial dysfunction has been shown to play a critical role in regulating inflammatory response of this disease.^9^, ^10^

Mitochondria are dynamic organelles undergoing continual fusion and fission events.^11^, ^12^ These dynamic changes modulate reactive oxygen species (ROS) generation, mitochondrial transport, cellular ion flow, and energy supply, thereby impacting a wide range of cellular processes.^13^, ^14^ In mammalian cells, mitochondrial fission is primarily mediated by dynamin‐related protein 1 (Drp1).^12^, ^15^ Upon activation by endogenous or exogenous stimulation, Drp1 accumulates in the mitochondrial outer membrane, leading to mitochondrial fragmentation and further ROS generation.^16^, ^17^ Emerging evidence suggests that overactivation of Drp1 and mitochondrial fragmentation may play a role in progression of allergic diseases,^18^, ^19^, ^20^ perturbing the normal functions of T cells, mast cells, and NK cells.^18^, ^21^ ROS derived from mitochondrial disfunction have been proposed to promote Th2 polarization, impaired the IFN‐γ expression, and heightened allergic responses through activation of JAKs and STAT6.^10^, ^22^, ^23^ However, the connection between mitochondrial fission and AR remains poorly understood.

Mitochondrial division inhibitor 1 (Mdivi‐1) is a selective inhibitor of mitochondrial fission via the Drp1 pathway. It has exhibited a favorable therapeutic effect for a wide range of pathological conditions including cardiovascular, nervous system, and autoimmune diseases, and shown to notably ameliorate the allergic inflammation in atopic dermatitis and asthma.^24^, ^25^ In the present study, we prepared a classic murine model of AR by sensitization with ovalbumin (OVA). Then, we explored the mitochondrial morphology in mice with AR, and evaluated the effects of Mdivi‐1 on allergic symptoms, inflammatory responses, and related molecules. Our results demonstrated that mitochondrial fragmentation is involved in OVA‐induced AR murine model, and inhibition of mitochondrial fission by Mdivi‐1 can alleviate allergic responses and related signals in this model, which suggests that mitochondrial fission may be a promising target for AR therapy.

## 2 MATERIALS AND METHODS

### 2.1 Animals

A total of 30 male‐specific pathogen‐free 6‐week‐old C57BL/6 mice weighing approximately 20 g were provided by Qinglongshan Experimental Animal Breeding Center. Animals were housed in a temperature‑controlled room (23 ± 2°C) at 55 ± 10% relative humidity with a 12‑h light/dark cycle and allowed free access to water and food. All animal experiments were performed in compliance with NIH guideline for the care and use of laboratory animals and approved by the Animal Ethics Committee of Nanjing University of Traditional Chinese Medicine (Approval Number: 201812A001, Nanjing, China).

### 2.2 Murine AR model and treatments

Mice were randomly divided into three groups by referring to a random numbers table: sham (*n* = 10), vehicle (*n* = 10), and Mdivi‐1 (*n* = 10). The method to induce AR model was modified from the previous studies.^26^, ^27^, ^28^ Briefly, mice were sensitized by intraperitoneal injection with 25 μg of OVA (Grade V, Sigma‐Aldrich) and 1 mg of aluminum hydroxide (Sinopharm Chemical Reagent Co., Ltd.) on Days 0, 7, and 14.^26^, ^28^ Subsequently, intranasal challenge was performed by intranasal instillation of 500 μg OVA dissolved in 20 μL of sterile saline (10 μL/nostril) using a micropipette from Days 21–27.^27^, ^28^ Mice in the sham group were treated with saline during the sensitization and challenge. Animals were excluded for the following reasons: serious infection, unstable vital signs, and/or death during experiment. No animals were excluded during this study.

**Figure 1:**
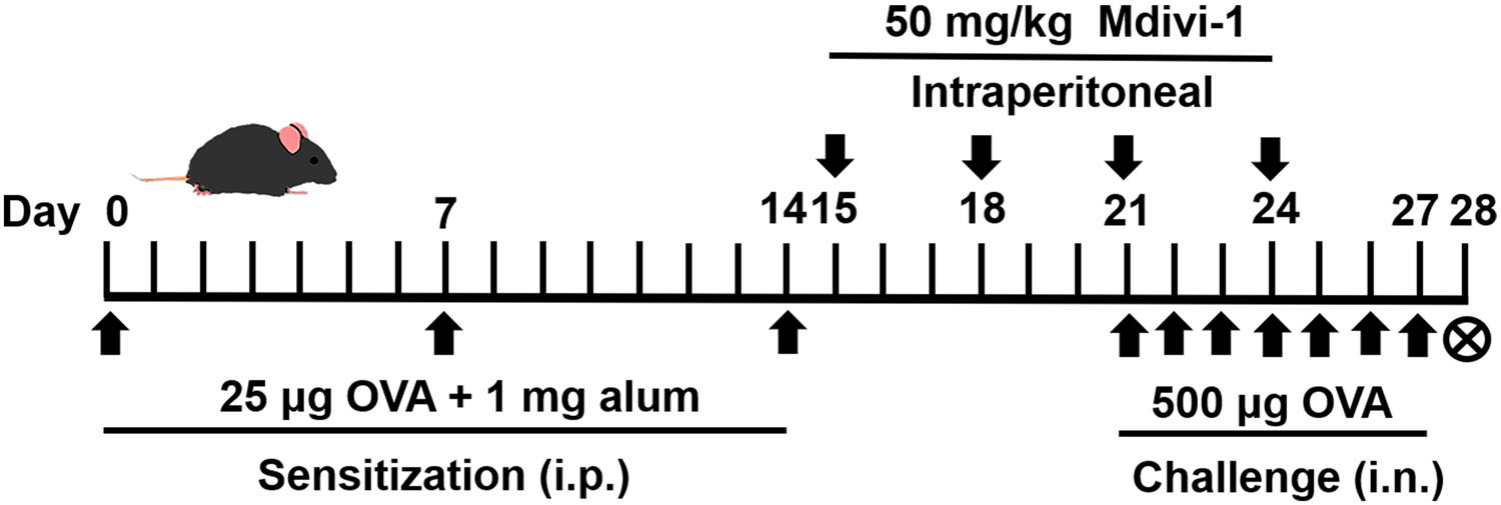
Flowchart of the ovalbumin (OVA)‐induced allergic rhinitis (AR) mouse model and treatment with mitochondrial division inhibitor 1 (Mdivi‐1).

Mdivi‐1 was obtained from Sigma‐Aldrich. After dissolving in dimethyl sulfoxide, Mdivi‐1 was diluted with sterile saline to 2.5 mg/mL. Mice in the Mdivi‐1 group were intraperitoneally administered Mdivi‐1 (50 mg/kg) on Days 15 and 18 as well as 1 h before intranasal challenge with OVA on Days 21 and 24. Animals in the sham and vehicle groups were treated with vehicle (saline containing 0.1% dimethyl sulfoxide). The schedule for allergen sensitization and treatment administration was summarized in Figure 1.

### 2.3 Evaluation of nasal allergic symptoms

On Days 21, 23, 24, and 27, mice were observed in blinded fashion for frequencies of nasal rubbing and sneezing in the 15‐min interval following intranasal OVA challenge.

### 2.4 Cytokine analysis in serum and nasal lavage fluid (NALF)

Twenty‐four hours after the last OVA challenge, mice were anesthetized with sodium pentobarbital and blood was collected from the orbital venous plexus. Serum was obtained by centrifugation at 1000 × *g*, 4°C for 10 min and stored at −80°C. Following sacrifice, the nasal cavities were immediately perfused with 1 mL of saline. The obtained NALF were centrifuged and supernatants were stored at −80°C until use. Concentrations of interleukin (IL)‐4, IL‐5, IFN‐γ, and OVA‐specific IgE in serum and NALF supernatants were assayed using enzyme‐linked immunosorbent assay (ELISA) kits (MultiSciences Biotech Co., Ltd; BioLegend).

### 2.5 Tissue processing and histopathology

Fresh nasal mucosa delaminated from nasal cavities were washed with phosphate‐buffered saline (PBS) and embedded to generate sections for ROS detection using fluorescent probe. The remaining heads were fixed in 4% paraformaldehyde for 3 days, then decalcified in ethylenediamine triacetic acid solution for another 5 days. After cryoprotection in a series of 20% and 30% sucrose solutions (Sigma‐Aldrich), samples were embedded in Optimal Cutting Temperature compound (Tissue‐Tek) and 7‐µm‐thick coronal sections were prepared. Frozen sections were stored at −80°C.^29^ Hematoxylin and eosin staining was conducted to assess inflammatory responses in nasal mucosa. Epithelial mast cell infiltration and percentage mast cell degranulation were assessed by toluidine blue staining.

### 2.6 Transmission electron microscopy

Nasal septal mucosa harvested from fixed heads were immersed in glutaraldehyde (2.5%) at 4°C overnight. Following several rinses with PBS, samples were postfixed with 2% osmic acid for 90 min, then dehydrated in a series of graded ethanol solutions. After infiltration in resin overnight, the mucosa was embedded and 70‐nm‐thick sections were prepared. After staining with 4% uranyl acetate (15 min) and 0.5% lead citrate (5 min), mucosal ultrastructure was scrutinized by transmission electron microscopy (Carl Zeiss). Mitochondrial length was measured with Image‐Pro Plus 6.0 software (Media Cybernetics) using the curve tracking function.

### 2.7 Immunofluorescence staining

Sections of nasal tissue were rinsed and blocked with 5% bovine serum albumin and 0.3% Triton X‐100 in PBS. Subsequently, sections were incubated with antibodies against p‐JAk2, p‐STAT6, TOM20 (all 1:200, Abcam), and p‐Drp1 (Ser616) (1:400, Cell Signaling Technology). Following rinses with PBS and incubation with Alexa 594‐conjugated secondary antibodies, slides were viewed under a fluorescence microscope (Olympus) or confocal fluorescence microscopy (Carl Zeiss; Leica).

### 2.8 Intracellular ROS measurement

Dihydroethidium (DHE; KeyGen Biotech), a ROS‐sensitive fluorescent probe, was used to detect intracellular ROS in nasal mucosa. Briefly, sections were incubated with 5 µM DHE for 30 min at 37°C in a dark room.^30^ After washing with PBS, fluorescence was visualized using a fluorescence microscope (Olympus).

### 2.9 Statistical analysis

Statistical analysis was carried out using Prism7 (GraphPad Software). The distribution of data was verified using the Shapiro–Wilk test. Differences between two groups were assessed using Student's *t*‐tests and differences among multiple groups were analyzed by one‐way or two‐way analysis of variance with Tukey's post‐hoc test. Data that were not normally distributed were analyzed using the nonparametric Mann–Whitney *U* test. Data were presented as means ± standard deviations (SD). Values of *p* < .05 were considered statistically significant.

## 3 RESULTS

### 3.1 Excessive mitochondrial fission is induced by OVA stimulation in a mouse model of AR

**Figure 2:**
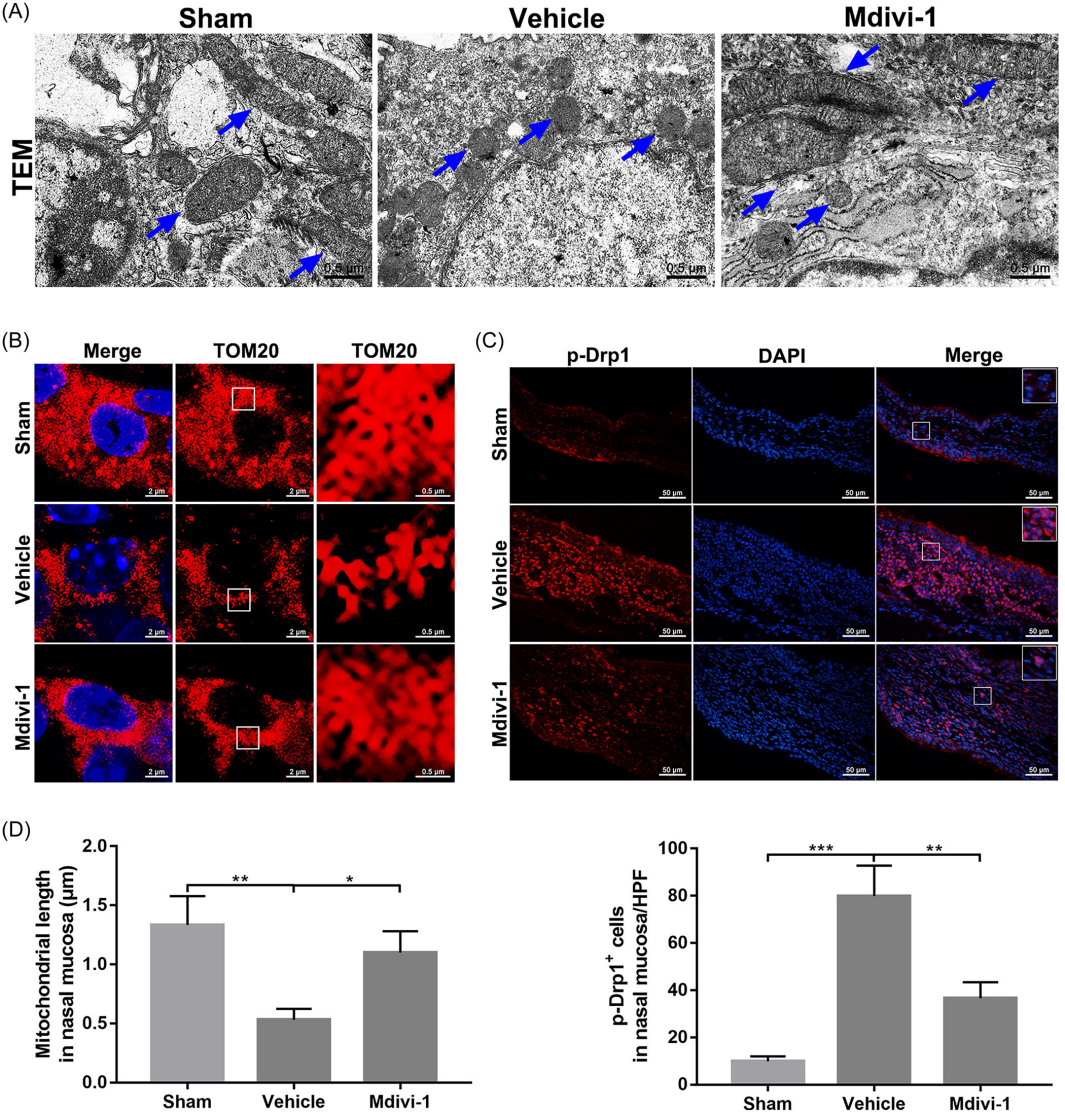
Mitochondrial fragmentation events were induced in ovalbumin (OVA)‐stimulated mice. (A) Mitochondrial morphology viewed by transmission electron microscopy in nasal mucosa (*n* = 3 mice per group; scale bar: 0.5 μm). (B) Mitochondrial imaging by TOM20 staining in nasal mucosa, the right end panels show magnified images (6.25×) of the regions indicated by white squares in the middle panels, the middle panels showed the pure TOM20 channel of the left end panels (*n* = 4 mice per group; scale bar: 2 μm in left and middle panels and 0.5 μm in right panels). (C) Immunofluorescence staining of p‐Drp1 (Ser616) in nasal mucosa (*n* = 4 mice per group; scale bar: 50 μm). (D) Quantitative analysis for mitochondrial length and immunofluorescence staining of p‐Drp1 (Ser616) in nasal mucosa. Statistics: one‐way analysis of variance with Tukey's multiple comparison correction. *p < .05, **p < .01, and ***p < .001.

Mitochondrial fragmentation has been demonstrated to participate in a variety of diseases.^11^, ^19^, ^31^, ^32^ However, the mitochondrial dynamics in AR remains obscure. Using transmission electron microscopy, we found that the ultrastructure of the nasal mucosa in sham group mice had a long tubular mitochondrial morphology with globular mitochondrial structures. These were converted into small, globular mitochondrial structures in the vehicle group following OVA stimulation, while Mdivi‐1 treatment appeared to prevent these morphological changes (Figure 2A,D). Consistently, TOM20 (a specific mitochondrial marker) staining of mitochondria confirmed the remarkable changes of the mitochondrial morphology in AR mice and the reverse effect of Mdivi‐1 on mitochondrial fragmentation events (Figure 2B).

Drp1 acts as the primary regulator of mitochondrial fission events.^15^ Phosphorylation of Drp1 at serine 616 facilitates mitochondrial translocation of Drp1, leading to fission events.^33^ Interestingly, our immunofluorescence analysis revealed an increase of Drp1 phosphorylation at serine 616 in the nasal mucosa following OVA stimulation, and this event was markedly suppressed after Mdivi‐1 intervention (Figure 2C,D). Thus, aberrant mitochondrial dynamics were involved in mice with AR, and Mdivi‐1 inhibited this mitochondrial fragmentation phenomenon.

### 3.2 Mdivi‐1 alleviated nasal allergic symptoms in a mouse model of AR

**Figure 3:**
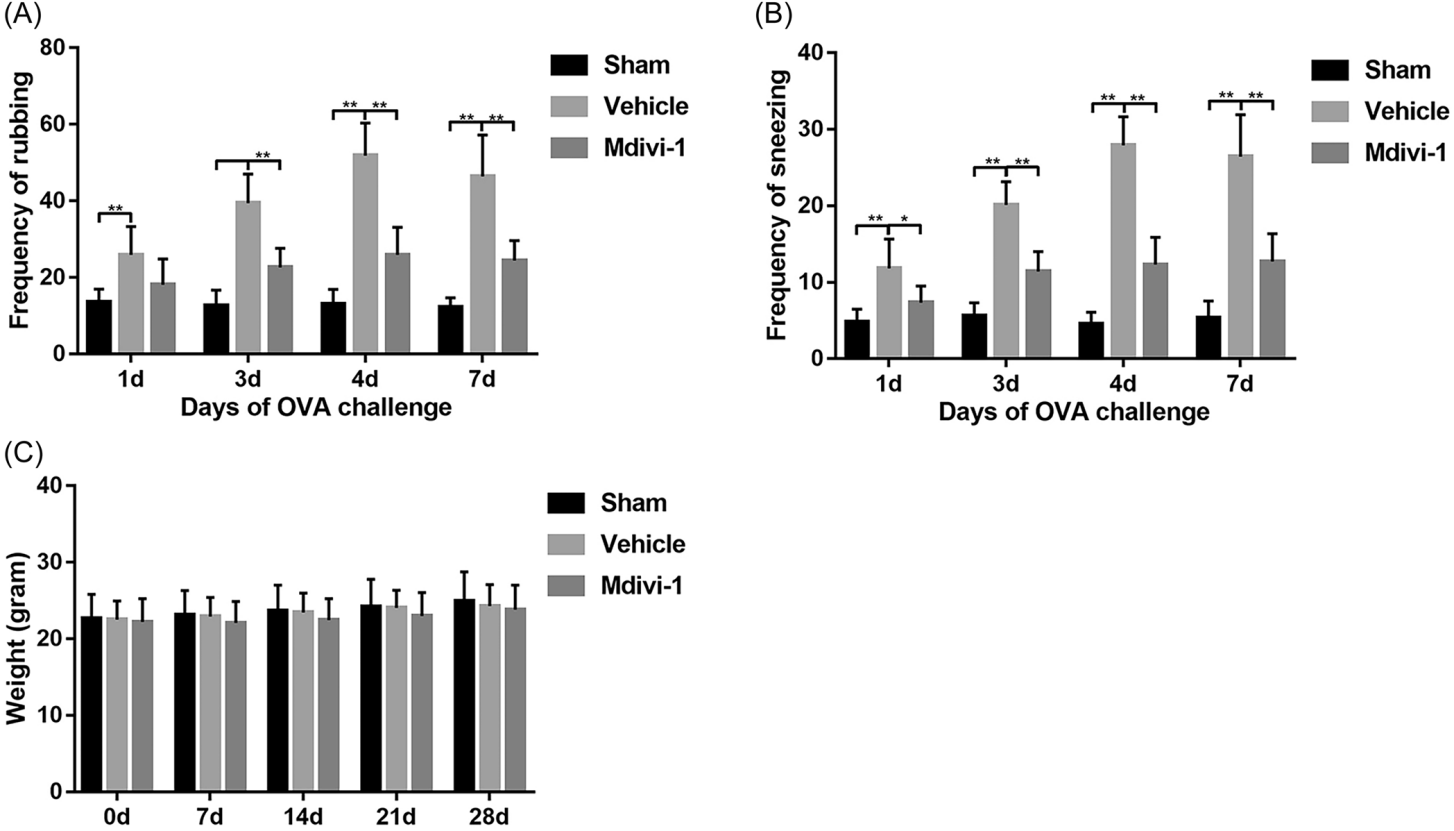
Mitochondrial division inhibitor 1 (Mdivi‐1) ameliorated nasal symptoms in mice with allergic rhinitis (AR). (A, B) Frequency of total rubbing and sneezing evaluated during the 15‐min interval after the intranasal ovalbumin (OVA) challenge (*n* = 10 mice per group). (C) Body weight in each group over time (*n* = 10 mice per group). Data are shown as mean ± SD. Statistics: two‐way analysis of variances with Tukey's multiple comparison correction. *p < .05, **p < .01, and ***p < .001.

As shown in Figure 3A,B, intranasal OVA challenge induced rubbing and sneezing responses, with frequencies of rubbing and sneezing generally increased following repeated OVA stimulation. The frequencies of nasal rubbing and sneezing were significantly reduced in mice treated with Mdivi‐1 compared with vehicle‐treated mice on Days 3, 4, and 7 of intranasal OVA challenge (Figure 3A,B). However, the body weights of mice showed no differences among the three groups over the entire period of stimulation and treatment (Figure 3C). Collectively, these results suggested that inhibition of mitochondrial fission by Mdivi‐1 ameliorated OVA‐induced allergic nasal symptoms.

### 3.3 Mdivi‐1 modulated allergic inflammatory responses in a mouse model of AR

**Figure 4:**
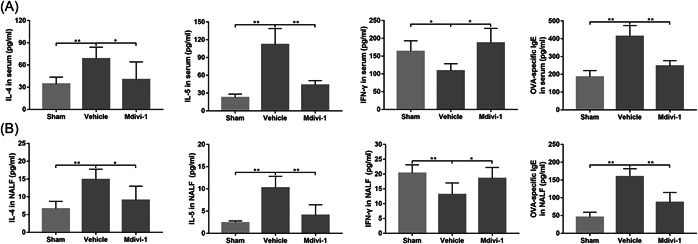
Mitochondrial division inhibitor 1 (Mdivi‐1) modulated cytokines in serum and nasal lavage fluid (NALF) in mice with allergic rhinitis (AR). (A, B) The levels of interleukin (IL)‐4, IL‐5, IFN‐γ, and ovalbumin (OVA)‐specific IgE determined by ELISA in serum (A) and NALF (B). Data are shown as mean ± SD (*n* = 6 mice per group). Statistics: one‐way analysis of variances with Tukey's multiple comparison correction. *p < .05, **p < .01, and ***p < .001.

Imbalances of Th1 and Th2 cytokines critically impact levels of allergen‐specific IgE and further development of allergic inflammation.^34^ Using ELISA, we observed decreased levels of IFN‐γ and increased expression of IL‐4 and IL‐5 in OVA‐sensitized mice. Consistently, expression of OVA‐specific IgE was also significantly increased in mice following OVA stimulation. However, Mdivi‐1 treatment notably suppressed production of IL‐4, IL‐5, and specific IgE following OVA stimulation and partly restored the IFN‐γ levels in both serum and NALF (Figure 4A,B).

**Figure 5:**
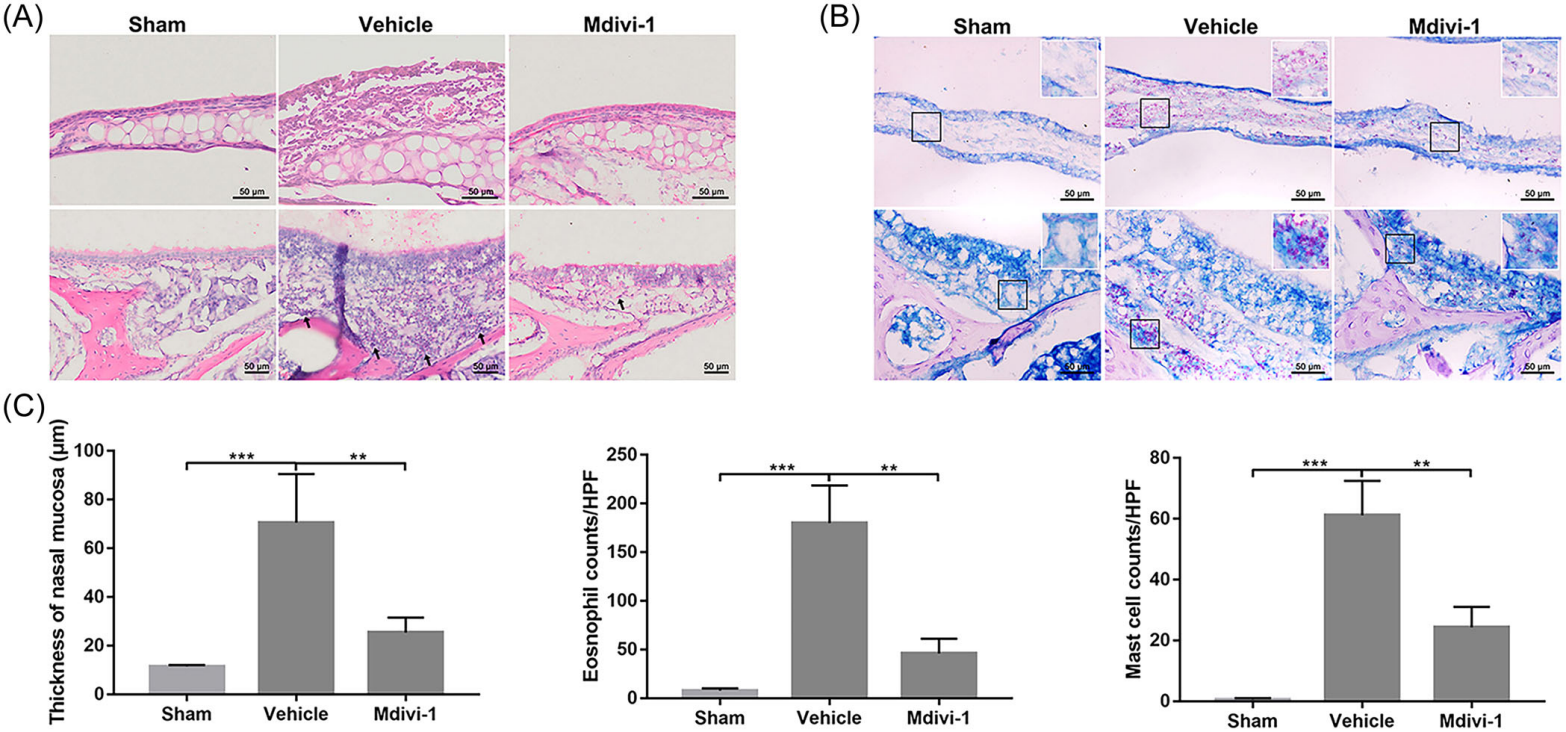
Mitochondrial division inhibitor 1 (Mdivi‐1) attenuated the allergic inflammatory response in nasal mucosa of ovalbumin (OVA)‐sensitized mice. (A) Hematoxylin/eosin staining of nasal mucosa for detecting the thickness of nasal mucosa and infiltration of eosinophils. Black arrow, eosinophils. (B) Toluidine blue staining of nasal mucosa for assessing the infiltration of mast cells. (C) Quantitative analysis of mucosa thickness and infiltration of eosinophils and mast cells. Scale bar: 50 μm. Data are shown as mean ± SD (*n* = 4 mice per group). Statistics: one‐way analysis of variances with Tukey's multiple comparison correction. *p < .05, **p < .01, and ***p < .001.

Hematoxylin/eosin staining revealed that OVA sensitization induced infiltration of eosinophils into the nasal mucosa and hypertrophy of the inferior turbinate; both phenotypes were partially prevented by Mdivi‐1 treatment (Figure 5A,C). Furthermore, Mdivi‐1 administration also markedly reduced the numbers of mast cells in the nasal mucosa of OVA‐sensitized mice (Figure 5B,C). Collectively, these results indicated that Mdivi‐1 blunted allergic inflammatory responses in a mouse model of AR.

### 3.4 Mdivi‐1 suppressed activation of JAK2/STAT6 and ROS generation in a mouse model of AR

**Figure 6:**
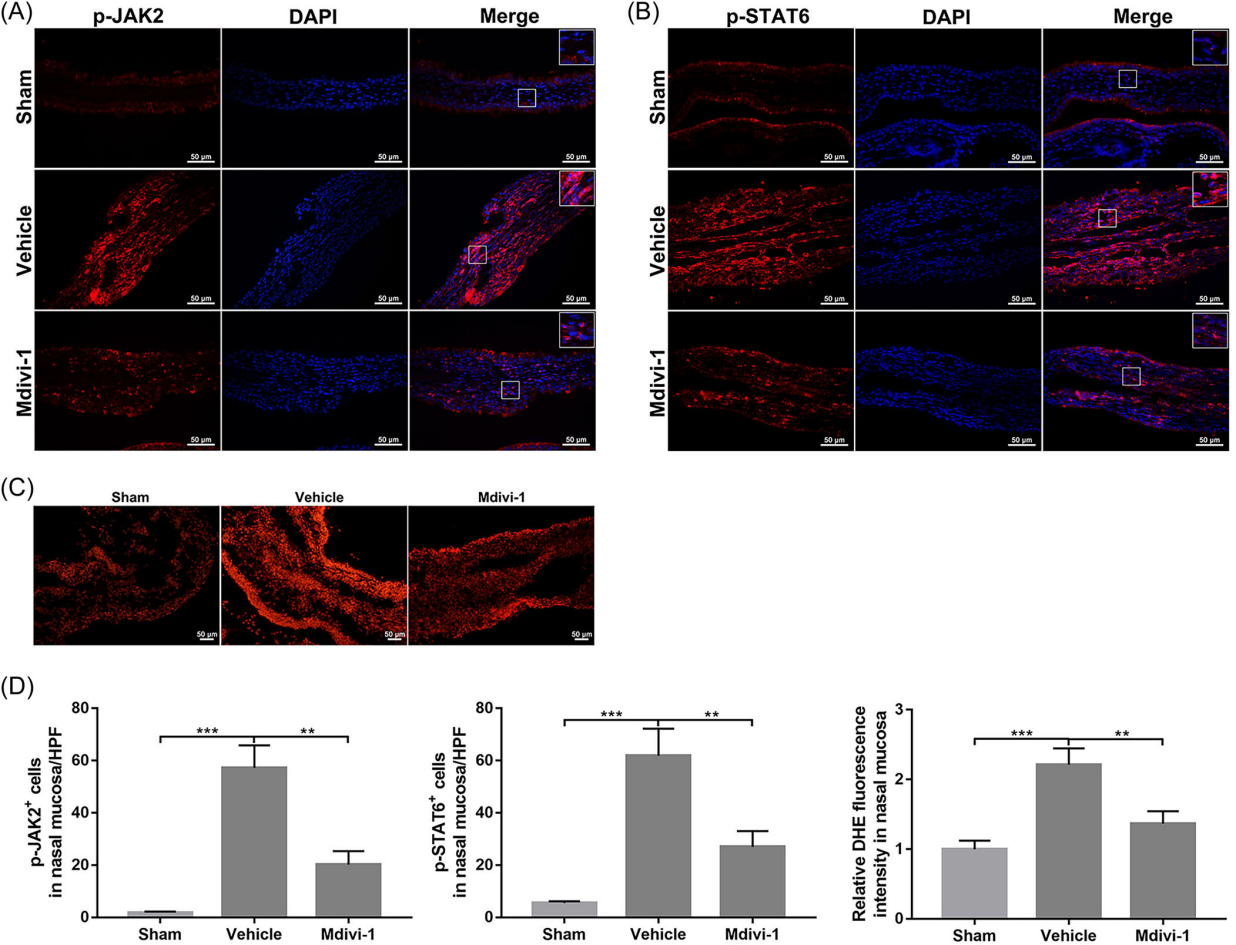
Mitochondrial division inhibitor 1 (Mdivi‐1) suppressed activation of JAK2 and STAT6 and reduced reactive oxygen species (ROS) generation in mice subjected to allergic rhinitis (AR). (A, B) Immunofluorescence staining of p‐JAK2 (A) and p‐STAT6 (B) in nasal mucosa (*n* = 4 mice per group. (C) Dihydroethidium (DHE) staining of nasal mucosa for detecting the level of intracellular ROS (*n* = 3 mice per group). (D) Quantitative analysis of immunofluorescence staining and DHE staining. Scale bar: 50 μm. Data are shown as mean ± SD. Statistics: one‐way analysis of variances with Tukey's multiple comparison correction. *p < .05, **p < .01, and ***p < .001.

Activation of JAK2 and STAT6 signals plays a critical role in regulating allergic inflammatory responses.^35^, ^36^ As shown in Figure 6, OVA sensitization was associated with increased counts of p‐JAK2^+^ and p‐STAT6^+^ cells in nasal mucosa. However, Mdivi‐1 treatment markedly reduced the levels of p‐JAK2 and p‐STAT6 in these tissues (Figure 6A,B,D), indicating an impact of mitochondrial dynamics on those allergic signals.

ROS, the critical products of mitochondrial fission, are known to act as important regulators for JAK2 or STAT6 and allergic responses.^10^, ^23^, ^37^ DHE staining indicated that OVA stimulation significantly increased intracellular ROS levels in the nasal mucosa (Figure 6C,D). Interestingly, Mdivi‐1 treatment markedly reduced ROS generation in OVA‐sensitized mice (Figure 6C,D). Overall, we determined that Drp1‐related mitochondrial fission involved in the allergic inflammation during AR, and inhibition of mitochondrial fission by Mdivi‐1 could attenuate allergic responses by regulating ROS production and downstream signaling molecules.

## 4 DISCUSSION

Emerging studies have implicated mitochondrial fragmentation in the progression of various allergic diseases^19^, ^38^; this process clearly impacts the functions of dendritic cells, T cells, and mast cells.^39^, ^40^ The role of mitochondrial fission in AR is poorly understood. Our study bridges this gap by examining the role of mitochondrial fission inhibition by Mdivi‐1 in modulating allergic inflammatory responses during AR. Our results indicate than restoring normal mitochondrial dynamics may be a promising strategy for controlling allergic inflammation and treating AR.

The balance between mitochondrial fusion and fission events is important for stabilizing normal cellular functions.^14^, ^40^ Excessive mitochondrial fission can cause mitochondrial dysfunction and enhanced generation of ROS,^17^ participating or aggravating the pathological changes in asthma and atopic dermatitis.^24^, ^25^, ^41^ In our study, results of transmission electron microscopy suggested increased frequencies of small, globular mitochondrial fragments in the nasal mucosa following OVA stimulation, implying a potential association between pathological changes of AR and mitochondrial fission. TOM20 is an outer mitochondrial membrane receptor involved in protein translocation and has been frequently used as specific marker of mitochondria.^42^, ^43^, ^44^ Consistently, TOM20 staining confirmed the enhanced mitochondrial fragmentation events in vehicle‐treated mice compared with the sham ones. Mdivi‐1, a specific inhibitor of mitochondrial fission via the Drp1 pathway,^45^, ^46^ has been widely applied in investigations of links between mitochondrial fragmentation and neurological disorders, cardiovascular diseases, and carcinogenic diseases.^46^, ^47^ Interestingly, the mitochondrial fragmentation events in nasal mucosa were also specifically reversed by Mdivi‐1 treatment in AR mice according to the combined results from transmission electron microscopy and TOM20 staining, verifying that Mdivi‐1 could act as a tool for exploring the connection of mitochondrial fission and this disease.

Nasal symptoms and allergic inflammatory responses are critical outcomes for evaluating effects of potential therapies on AR.^26^ Mdivi‐1 application reduced the frequencies of rubbing and sneezing in mice following OVA stimulation. Moreover, increased levels of Th2 cytokines and specific IgE in the serum and NALF were notably downregulated after Mdivi‐1 treatment. The histopathologic staining confirmed the role of Mdivi‐1 treatment in modulating allergic inflammatory responses in mice with AR. The allergic responses during AR are highly related to JAK2 and STAT6 signaling,^48^, ^49^ which act as important downstream signals of mitochondrial fission and subsequent ROS generation.^16^ In our study, intracellular ROS generation in nasal mucosa were notably increased following OVA stimulation, while Mdivi‐1 partly reversed this pathological change. Then, activation of JAK2 and STAT6 in AR mice was also restrained by Mdivi‐1 treatment. In summary, these results indicated that mitochondrial fragmentation participated in the pathological events underlying AR, and restoring normal mitochondrial dynamics using Mdivi‐1 ameliorate the allergic symptoms and inflammatory responses in a murine model of AR.

Our study had several limitations. First, although Mdivi‐1 showed promising efficacy in ameliorating allergic responses in AR, we did not assess the safety or optimal dosing of this agent. These factors may impact the clinical translation of this molecule. Second, Drp1‐deficient mice, were not used in our study because of technical difficulties, limiting our ability to more accurately understand the relationships between mitochondrial dynamics and AR. An inactivate form of Mdivi‐1 was not used in our study, and thus we cannot be certain that the drug did not have off target effects that may have been partially responsible for the observed results. Third, in vitro experiments assessing the connection between mitochondrial dynamics and functions of related immune cells during AR were not conducted in our study. This provides a meaningful focus for future research and we plan to address some of these issues in our upcoming work.

## 5 CONCLUSION

In conclusion, aberrant mitochondrial dynamics play important roles in allergic responses in AR. Inhibition of mitochondrial fragmentation by Mdivi‐1 can ameliorate the allergic symptoms and inflammatory responses in a murine model of AR, suggesting that restoring normal mitochondrial dynamics may be a promising strategy for treatment of AR.

## AUTHOR CONTRIBUTIONS

**Xu‐qing Chen**: Conceptualization; data curation; formal analysis; methodology; writing—original draft. **Long‐yun Zhou**: Data curation; formal analysis; methodology; visualization; writing—original draft. **Hua‐an Ma**: Investigation; validation. **Ji‐yong Wu**: Investigation; validation. **Shu‐fen Liu**: Investigation; validation. **Yong‐jun Wu**: Conceptualization; supervision; writing—review and editing. **Dao‐nan Yan**: Conceptualization; supervision; writing—review and editing.

## CONFLICT OF INTEREST STATEMENT

The authors declare no conflict of interest.

## ETHICS STATEMENT

This study was approved by the Animal Ethics Committee of Nanjing University of Traditional Chinese Medicine (Approval Number: 201812A001, Nanjing, China).

## Data Availability

Data are available from the corresponding author upon reasonable request.
